# The Samuel D. Gross Professorship of Pathological Anatomy

**Published:** 1884-09

**Authors:** 


### EDITORIAL.

---

THE SAMUEL D. GROSS PROFESSORSHIP OF PATHOLOGICAL ANATOMY.

We call attention to the appeal, in another column, to secure in some medical school the endowment of a *memorial professorship*, to be designated the "S. D. Gross Professorship of Pathological Anatomy."

Extended comment upon this subject is rendered unnecessary by the nature of the case. The gifted editor of *The American Journal of the Medical Sciences*, in the July number, current year, of that journal, has well said in the closing lines of his eloquent memoir upon "The Nestor of American Surgery":—

"In the death of Dr. Gross we have lost one of the brightest examples of the skill and learning, the conscientiousness and assiduity, the patience and perseverance, the dignity and morality by which our profession is truly ennobled. He has left us as a heritage a world-wide reputation which, as we regard it with conscious pride, cannot but stimulate us to a higher sense of duty to our profession and to our fellows."

The endowment of a *memorial professorship* is a peculiarly fitting and graceful recognition of one of the crowning glories of Professor Gross's life. He was, in the widest sense of the term, a great teacher. Entirely apart from his brilliant career as Professor of Surgery, his heroic and herculean efforts to elevate the standard of American Medical Education are fresh in all our memories. Great wisdom has been evinced in the selection of Pathological Anatomy as the subject of the *memorial professorship.* Professor Gross's pioneer researches in this field,—of whose topography, American and English surgeons, even at the present day, are only too ignorant,—are integral portions of the history of American Medicine and Surgery.

The medical school, in which the endowment shall be secured, is not mentioned in the appeal. We trust, however, the Jefferson Medical College will be chosen. For twenty-eight years the welfare of this institution was among the most ardently cherished purposes of the great surgeon's life.

The jealous guarding of the *esprit de corps* of his own profession, the deep interest in the struggles and success of young men, the wide and generous hospitality of his house were characteristics, which endeared Professor Gross to the members of the medical profession, and rendered his name a household word. A touching instance of his cavalier-like disposition occurred a few months before his death. In November of last year, the medical profession was suddenly called to mourn the loss of that great and good man, J. Marion Sims. Professor Gross, in a letter appearing in the columns of the *New York Medical Record*, was *first* to suggest and *first* to contribute to the "Sims Memorial Fund."

On the ninth of June, a meeting of the medical profession of Philadelphia was called to take action upon the death of its illustrious member. At this meeting,—a thoroughly representative one,—a committee was appointed to issue the appeal, which appears in our columns. The names of the distinguished gentlemen,—composing this committee,—constitute an adequate guaranty of the purity of their motives. The Provost of the University of Pennsylvania and the Dean of the Faculty of the Jefferson Medical College are endeavoring to secure a common end.

Surely, under conditions like these, it is time to lay aside all petty party malice, spite and jealousy!

While the American medical mind is thus strongly moved by feelings of sorrow for its departed chieftain, opposition to the general will seems to be in peculiarly bad taste.

In the editorial columns of the *New York Medical Record*, 19th July, appears an acrimonious criticism of the proposed *memorial professorship*, in which desire to perpetuate the fame of Professor Gross, but unwillingness to contribute to the resources of "a private medical college," is expressed.

We are not at all surprised at this action of the *New York Medical Record.* It is merely a local expression of that thoroughly commercial spirit which has actuated this Journal to preserve silence upon the subject of higher medical education, and has instigated the support of the doctrine that medical gentlemen ought to meet in consultation with men whom they believe to be knaves or fools.

A profound student of medicine, in an essay, "Of Nature in Men," once said:—

"Nature is often hidden, sometimes overcome, seldom extinguished."

We are surprised, however, at the cool effrontery of *The Record*, when it ascribes a similarly sordid mode of thought and action to "many, perhaps most physicians." The cheerful alacrity, with which physicians, generally, and the eminent members of the profession in New York city, particularly, have responded and are responding, is a sufficient refutation of this remarkable assertion.

As the school in which the Chair shall be endowed has not been named, as yet, *The Record's* ill-natured remark is purely a work of supererogation. But the allusion to the Jefferson Medical College as "a private medical school" is untruthful. *The Record's* idea of American Medical Schools seems to be based upon the conception which is current among certain members of the profession in New York city. "What is an American Medical College?" asked a celebrated New York gynæcologist, very recently. "An association of doctors, who want to make a little money." If we were to judge from the attitude of *The Record* upon this and kindred subjects, and if we were to admit that *The Record* is, in any degree, exponential of the views of the local profession, we would be compelled to believe that the distinguished ex-professor's *dictum* expresses a grave truth, so far as the New York Medical Schools are concerned.

